# Using Telerehabilitation to Deliver a Home Exercise Program to Youth With Arthrogryposis: Single Cohort Pilot Study

**DOI:** 10.2196/27064

**Published:** 2021-07-06

**Authors:** Marianne Gagnon, Gabriela Marino Merlo, Rita Yap, Jessica Collins, Caroline Elfassy, Bonita Sawatzky, Jacquelyn Marsh, Reggie Hamdy, Louis-Nicolas Veilleux, Noémi Dahan-Oliel

**Affiliations:** 1 Shriners Hospitals for Children-Canada Montreal, QC Canada; 2 Department of Surgery McGill University Montreal, QC Canada; 3 School of Physical and Occupational Therapy McGill University Montreal, QC Canada; 4 Department of Orthopedics University of British Columbia Vancouver, BC Canada; 5 School of Physical Therapy Western University London, ON Canada; 6 Division of Paediatric Orthopaedics Department of Paediatric Surgery Montreal Children Hospital Montreal, QC Canada

**Keywords:** telerehabilitation, teleassessment, arthrogryposis multiplex congenita, physical therapy, occupational therapy

## Abstract

**Background:**

Arthrogryposis multiplex congenita (AMC) is characterized by joint contractures and muscle weakness, which limit daily activities. Youths with AMC require frequent physical therapeutic follow-ups to limit the recurrence of contractures and maintain range of motion (ROM) and muscle strength; however, access to specialized care may be limited because of geographical distance. Telerehabilitation can offer a potential solution for delivering frequent follow-ups for youth with AMC, but research on the use of telerehabilitation in children with musculoskeletal disorders is scarce.

**Objective:**

The study aims to evaluate the feasibility of delivering a home exercise program (HEP) by using telerehabilitation for youth with AMC. We also aim to explore the effectiveness of the HEP as a secondary aim.

**Methods:**

Youths aged between 8 and 21 years with AMC were recruited at the Shriners Hospitals for Children–Canada. The participants completed baseline and post-HEP questionnaires (the Physical Activity Questionnaire for Adolescents, Pediatrics Outcomes Data Collection Instrument, and Adolescent and Pediatric Pain Tool), and clinicians assessed their active ROM using a virtual goniometer. Clinicians used the Goal Attainment Scale with the participants to identify individualized goals to develop a 12-week HEP and assess the achievement of these goals. Follow-ups were conducted every 3 weeks to adjust the HEP. Data on withdrawal rates and compliance to the HEP and follow-ups were collected to assess the feasibility of this approach. The interrater reliability of using a virtual goniometer was assessed using the intraclass correlation coefficient and associated 95% CI. Nonparametric tests were used to evaluate feasibility and explore the effectiveness of the HEP.

**Results:**

Of the 11 youths who were recruited, 7 (median age: 16.9 years) completed the HEP. Of the 47 appointments scheduled, 5 had to be rescheduled in ≤24 hours. The participants performed their HEP 2.04 times per week (95% CI 1.25-4.08) and reported good satisfaction with the approach. A general intraclass correlation coefficient of 0.985 (95% CI 0.980-0.989) was found for the web-based ROM measurement. Individualized goals were related to pain management; endurance in writing, standing, or walking; sports; and daily activities. In total, 12 of the 15 goals set with the participants were achieved. Statistically significant improvements were observed in the pain and comfort domain of the Pediatrics Outcomes Data Collection Instrument (preintervention: median 71; 95% CI 34-100; postintervention: median 85; 95% CI 49-100; *P*=.08) and Physical Activity Questionnaire for Adolescents (preintervention: median 1.62; 95% CI 1.00-2.82; postintervention: median 2.32; 95% CI 1.00-3.45; *P*=.046).

**Conclusions:**

The remote delivery of an HEP for youth with AMC is feasible. Promising results were found for the effectiveness of the HEP in helping youths with AMC to achieve their goals. The next step will be to assess the effectiveness of this exercise intervention in a randomized controlled trial.

**International Registered Report Identifier (IRRID):**

RR2-10.2196/18688

## Introduction

### Background

**Figure 1:**
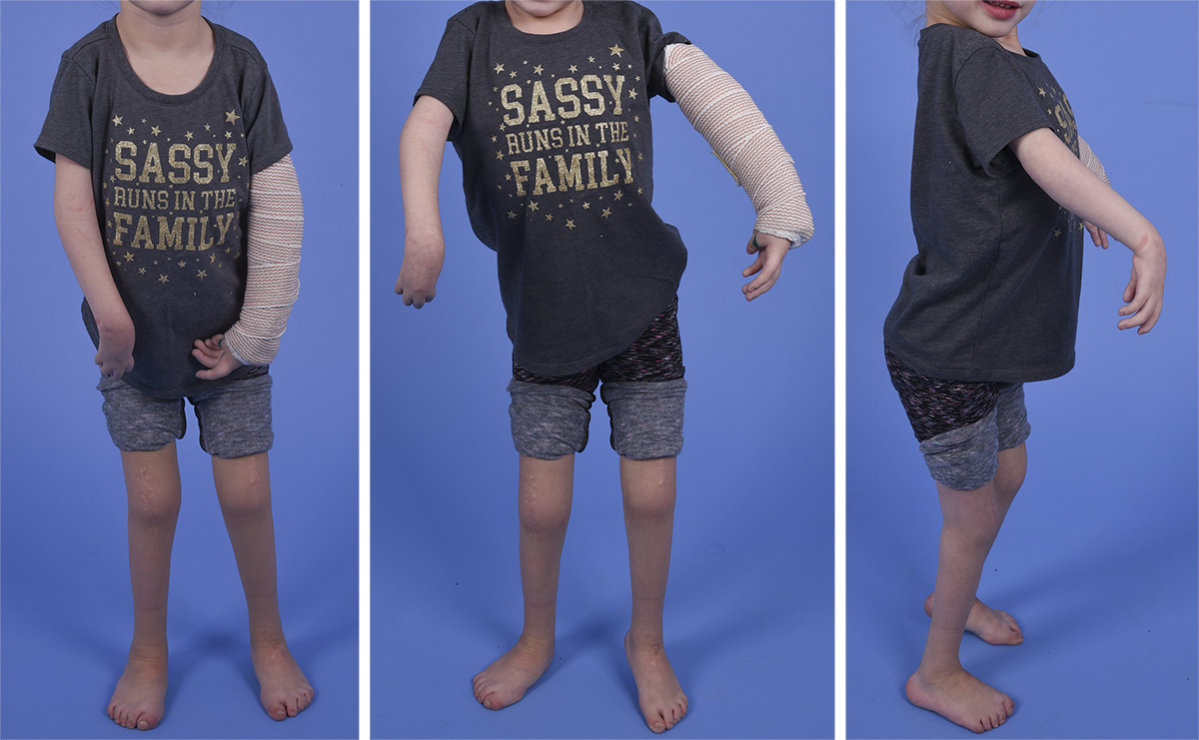
Young female with Amyoplasia with typical positioning of the limbs (ie, internal rotation at the shoulders, extension contractures at the elbow, and flexion contractures at the knees).

**Figure 2:**
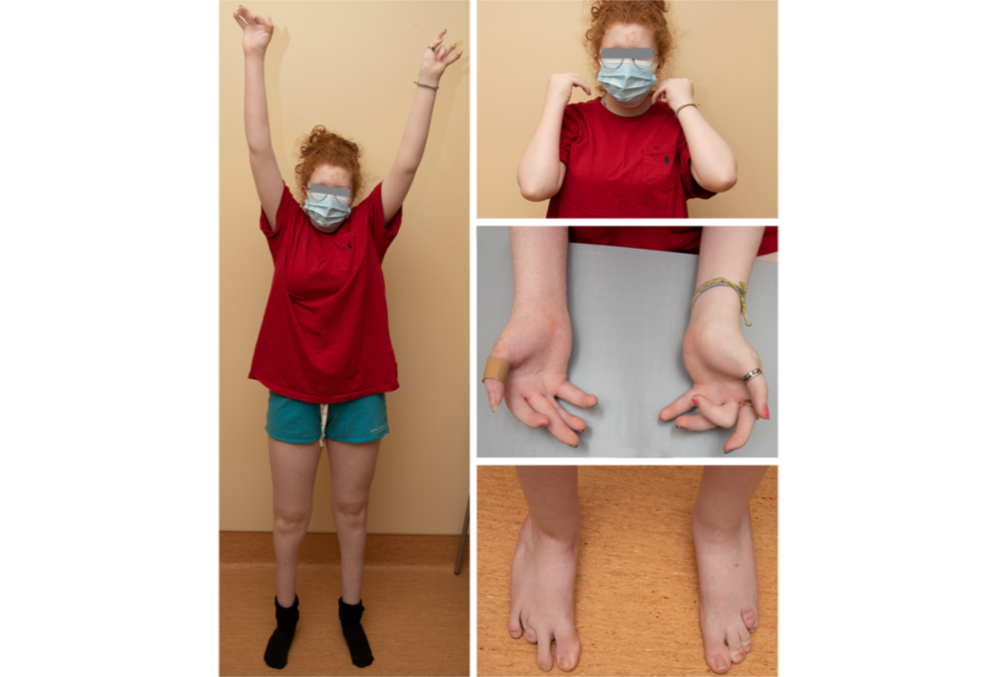
Adolescent with distal arthrogryposis, good range of motion at the shoulders and elbows (shoulder flexion and elbow flexion), and typical hand and foot deformities.

Arthrogryposis multiplex congenita (AMC) is an umbrella term used to describe more than 400 conditions characterized by congenital joint contractures present in at least two body areas and affecting 1 in 4300-5100 live births [1,2]. Amyoplasia and distal arthrogryposis are the most common types of AMC, representing approximately 50%-65% of all AMC diagnoses [3,4]. Amyoplasia is characterized by decreases muscle mass and the typical positioning of the limbs (Figure 1) [5,6]. Distal arthrogryposis is characterized by contractures that mainly affect the distal limbs (ie, feet and hands; Figure 2) [6]. Individuals with AMC may have poor muscle mass in addition to limitations in range of motion (ROM), resulting in difficulties in transfers, mobility, and independent activities of daily living [6-8]. One aspect contributing to the limited ROM in individuals with AMC is the lack of movement of limbs, which may be overcome by physical exercise [6]. Some studies speculated that exercise in youth with AMC may maintain ROM, increase muscle strength, and decrease pain, but the direct effects of an exercise program remain to be tested in this population [3,6,9,10]. A positive association was also reported between knee and hip muscle strength and motor function (eg, rolling, sitting, standing, and climbing) in individuals with AMC, suggesting the importance of preserving sufficient muscle strength for daily activities [11]. Currently, most interventions in individuals with AMC, specifically rehabilitation, occur in early childhood, and the frequency decreases during school-age and adolescent years despite new challenges arising during these transition periods [12,13]. Rehabilitation for school-aged children with AMC focuses mostly on body functions (eg, mobility of joints and muscle endurance) and structure (eg, joint contracture of the elbow or knee), which does not always correspond to the individual’s specific needs such as participating in activities and increasing independence for attending school and employment [14].

### Rationale

Given the rarity, youths with AMC often require specialized care, but many of these individuals live far from subspecialized health care centers. Therefore, clinicians and researchers face challenges in developing adjunct therapies with frequent follow-ups, such as an exercise program. To overcome such challenges, novel intervention approaches and technologies are needed to increase access to subspecialized care for individuals with AMC living in remote areas. Telerehabilitation, an innovative way to deliver rehabilitation services remotely using telecommunication technologies, may offer a solution for delivering intervention programs with frequent follow-ups [15]. This new approach may contribute to reduced costs and decreased in-person visits to the hospital [16]. Telerehabilitation is not only useful for people with AMC living in remote areas but also useful for those living close by when they need consultations during a pandemic (such as the COVID-19 pandemic) in which social distancing is needed [17]. Despite its benefits, telerehabilitation has been understudied in children with physical disabilities [18]. Therefore, the primary aim of this pilot study is to evaluate the feasibility of using telerehabilitation to provide a home exercise program (HEP) for youth with AMC. As part of the feasibility objective, the interrater reliability of using a virtual goniometer for ROM measurements is explored. The secondary aim is to explore the effectiveness of this type of intervention.

## Methods

### Ethics and Recruitment

This prospective interventional single-cohort pilot study was conducted between January 2019 and March 2020 at the Shriners Hospitals for Children (SHC)–Canada. The study was approved by the department of medical research at the SHC (CAN1806) in July 2018, and ethics approval was received from the institutional review board of the McGill University Faculty of Medicine (#A08-B38-18B) in October 2018. Patients were included if they were aged between 8 and 21 years, understood written and spoken English or French, and had multiple congenital contractures as documented in their electronic medical records. Individuals were excluded if they had undergone a recent surgery (ie, 3 months prior for soft tissue and 6 months prior for bony surgery), lived outside Canada, or had cognitive deficits. Potential participants were identified by reviewing onsite medical records, and all eligible youths were approached during their clinic visit or by postal mail or phone if they did not have an upcoming appointment. Recruitment was also sought by posting an advertisement on a Canadian AMC support group on social media. Informed consent forms were filled by all parents and youths aged ≥14 years. Assent was provided for those aged 8-13 years.

### Assessment

To establish the 12-week HEP, an initial assessment was conducted by an occupational therapist and a physical therapist using a videoconferencing platform. This assessment included active ROM measurements of the upper and lower limb joints, an evaluation of overall function (mobility, transfers, and activities of daily living), and a pain assessment using the Adolescent and Pediatric Pain Tool (APPT). For the ROM assessment, the participants were asked to hold different positions, and screen captures were collected for subsequent measurements. The therapists guided the participants and the caregiver, when present, to ensure that the movements were performed properly and in the right plane of the camera (eg, the therapists asked for the camera to be tilted when needed). Based on the assessment and needs of the participants, the therapists and participants determined individualized goals using the Goal Attainment Scale (GAS). The scoring of the goals was defined according to the type of goal set, such as the percentage of perceived strength, a 0-10 satisfaction scale, or walking endurance measured in minutes. The information obtained through this initial assessment was used by the rehabilitation team to develop an individualized 12-week HEP. In addition, the participants were asked to complete web-based questionnaires, including the Physical Activity Questionnaire for Adolescents (PAQ-A) and the Pediatrics Outcomes Data Collection Instrument (PODCI), and to provide baseline disorder and sociodemographic information (ie, type of AMC, location of joint contractures, services care received, schooling, employment, volunteering, and leisure). All telerehabilitation meetings were conducted remotely using Zoom Pro (Zoom Video Communications Inc), a videoconferencing platform that allowed an encrypted connection and synchronous exchange between the participants and therapists. When needed, a parent was present to help position the camera and position or assist the participant or aid in the exercise material setup. In 1 case, the telerehabilitation meetings took place at the participant’s school with the assistance of the school therapist. The methods used for the pilot study are described in detail in Gagnon et al [19].

At the end of the 12-week HEP, the participants underwent a final assessment, with the occupational therapist and physical therapist using the same outcome measures as those used during the initial assessment, and they completed the same web-based questionnaires with the addition of 19 closed-ended and 4 open-ended questions on their satisfaction with the HEP.

### Intervention

**Figure 3:**
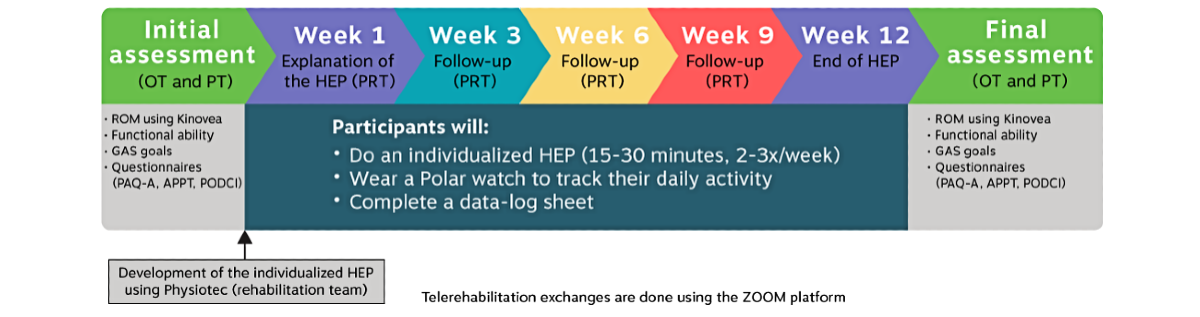
Timeline of the telerehabilitation intervention. APPT: Adolescent and Pediatric Pain Tool; GAS: Goal Attainment Scale; HEP: home exercise program; OT: occupational therapist; PAQ-A: Physical Activity Questionnaire for Adolescents; PODCI: Pediatrics Outcomes Data Collection Instrument; PRT: physical rehabilitation therapist; PT: physical therapist; ROM: range of motion.

All information gathered during the initial assessment was used to build an individualized HEP for each participant based on their goals and abilities. The therapists used Physiotec (Physiotec Québec Inc), a software program that easily creates an HEP and provides participants with access to detailed instructions and videos of the exercises. Examples of these HEPs can be found in the Multimedia Appendix 1. A week after the initial assessment, a physical rehabilitation therapist (PRT), a professional whose role is to develop treatment plans and provide appropriate intervention in collaboration with a physical therapist, explained the HEP to each participant during a remote session. When needed, the PRT demonstrated the exercises. During this session, the PRT ensured that the participant understood and performed the exercises safely. The participants were asked to perform their HEP 3 times a week for approximately 15 to 30 minutes at a time. Follow-ups were provided every 3 weeks (ie, at weeks 3, 6, and 9) by the PRT to ensure that the exercises were properly performed and to assess the level of difficulty of the HEP. Exercises were adjusted for progression or regression as needed (eg, number of repetitions and type of exercise). When necessary, for optimal loading, materials such as elastic resistance bands or theraputty (a silicone-based exercise material used for hand therapy) were sent to participants by postal mail. To record the compliance to the HEP, the participants were asked to wear a Polar A370 activity monitor (Polar Electro, Inc), which was sent to them by postal mail, to capture their exercise sessions and to complete a data log sheet. The activity monitor was worn on the wrist during the HEP session and was used to capture the number of exercise sessions. The participants were asked to charge their activity monitor every 3 to 4 days and, at the same time, to download their data to the Polar Flow platform through Bluetooth or direct connection. The downloaded data became available to the research team throughout the Polar Flow for Coach web platform. The research assistant (MG) manually scrutinized all recorded sessions on the Polar Flow for Coach web platform to remove those that were too short to be considered exercise sessions (≤15 min) or were recorded as another sport and made a note on the data log sheet (eg, hockey or skiing). Figure 3 shows a summary of the intervention.

### Statistical Analysis

#### Overview

Given that this was a pilot study with a small sample size and a heterogeneous population, nonparametric and descriptive statistics were used. *P* value corrections for multiple comparisons were not used because of the exploratory purpose of this study. Statistical analyses were performed using IBM SPSS Statistics version 24 for Windows (IBM Corp).

#### Feasibility

The feasibility was evaluated using different operationalization criteria. These operationalization criteria are listed in Textbox 1 and defined in detail in the published protocol for this study [19]. Compliance to the HEP and the telerehabilitation meetings were described using summary statistics and compared with pre-established feasibility criteria using a 1-sample Wilcoxon signed-rank test. For the remaining operationalization criteria, a comparison with pre-established criteria was performed using the same method. The technical issues (major technical issues being issues that resulted in the cancellation of the meeting and minor issues being something that could be resolved during the meeting) experienced during the program were also descriptively assessed using summary statistics. As part of evaluating feasibility, this pilot study also aimed to determine the most suitable outcome measures for this type of intervention.

**List of the operationalization criteria for assessing feasibility.**

**Source of recruitment**

- At clinic, postal mail, phone, and social media

**Recruitment rates**

- ≥50% of the eligible and reachable youths

**Withdrawal rates (before the intervention)**

- ≤20% of the youths who consent

**Withdrawal rates (during the course of the intervention)**

- ≤30% of the youths who start the intervention

**Completion rates**

- ≥50% of the youths who consent

**Compliance to the home exercise program**

- ≥50% of compliance to the home exercise program trainings

**Compliance to the telerehabilitation meetings**

- ≤15% of the meetings missed

**Missing data**

- ≤10% for each outcome

**Technical issues**

- Echo voices, connection, and image quality

#### Interrater Reliability

The interrater reliability of using a virtual goniometer to measure active ROM has been shown to be feasible when compared with an in-person assessment with a goniometer on 10 healthy adults [20]. However, its reliability has not been assessed in children and adolescents with a musculoskeletal disorder such as AMC. The interrater reliability was established by having 2 raters (MG and GMM) use a virtual goniometer (Kinovea version 0.8.15) to measure the ROM of 4 participants selected at random. ROM measurements taken in the appropriate plane of movement were included in the analysis. The intraclass correlation coefficient (ICC) and associated 95% CI for each joint and overall were calculated based on a single-measurement, absolute-agreement, two-way random-effects model [21-23].

#### Effectiveness

Summary statistics, including the median and 95% CI for continuous variables and counts and proportions for categorical variables, were produced for all variables. As this was a pilot study on a rare disorder, statistical comparisons were produced only for exploratory purposes using a significance (α) level set a priori to 10% as has been proposed for research on rare disorders [24].

The effectiveness of the HEP was explored using the GAS. Raw GAS scores were converted to GAS *t* scores for each participant and the global HEP [25]. Within-participant pre- and postintervention scores of the PODCI, PAQ-A, APPT, and ROM were compared using the related-samples Wilcoxon signed-rank test. Individual changes were also compared to evaluate clinically important differences as defined by Oeffinger et al [26] in the following domains of the PODCI (changes in points associated with a minimum clinically important difference): upper extremity (4.8 points), transfers and mobility (5.2 points), sports and physical function (10.3 points), pain and comfort (26.3 points), happiness (11.2 points), and global function (8.2 points). Changes in the APPT for the 10-point pain intensity scale were considered clinically meaningful when there was a change of more than two points [27,28].

For the PODCI and PAQ-A questionnaires, a sensitivity analysis was conducted because a participant had an ankle injury, unrelated to the HEP execution, before the final assessment. An imputation technique was used to replace the final results of the participant who was directly affected by the injury by using the median of the group for the PAQ-A and in the following domains of the PODCI: transfer and mobility, sport and physical function, pain and comfort, and global function.

## Results

### Participant Information

**Figure 4:**
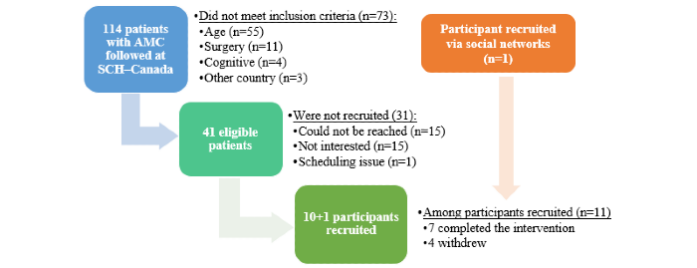
Participant flowchart. AMC: arthrogryposis multiplex congenita.

Of the 114 patients with AMC followed at the SHC–Canada, 26 (22.8%) were eligible to participate and were able to be approached for participation. Of these, 10 individuals consented to the study, and an additional participant not actively followed at the SHC was recruited on social media. A total of 7 participants (5 boys) completed the intervention. The participants were recruited at the clinic (n=6) or through postal mail (n=2), phone (n=2), or social media (n=1). Of the 4 participants who withdrew, 2 consented at the clinic but never started the HEP because they could not be reached to schedule the initial assessment, and 2 withdrew after the start of the HEP because of personal reasons and lack of time. Figure 4 shows the participation flow diagram. The participants’ median age was 16.9 years (range 11.3-20.8 years), and they represented 4 Canadian provinces with a median distance from the SHC–Canada of 227 km (range 7-3439 km). A total of 2 participants had Amyoplasia, 4 had distal arthrogryposis, and 1 had an unknown type of AMC. Joint involvement was distributed as follows: shoulders (n=5), elbows (n=6), wrists (n=6), hands (n=4), hips (n=3), knees (n=5), ankles (n=6), and spine (n=1), with a median of 6 joints (95% CI 3-8) involved. For the level of ambulation, 4 participants were classified as community ambulators, 2 were classified as household ambulators, and 1 was classified as nonambulatory.

### Feasibility

The recruitment rate among eligible participants followed at the SHC who were reachable was 38% (10/26), which was lower than the target set at ≥50%. The withdrawal rate before the start of the intervention was 18% (2/11) and after the start of the intervention, it was 18% (2/11), which met the established criteria of ≤20% and ≤30%, respectively. The HEP completion rate was 64% (7/11), which corresponded to the criterion of ≥50%. The number of meetings that needed to be rescheduled in ≤24 hours was 11% (5/47), which reached the target set at ≤15%. The median HEP compliance was 2.04 times per week (95% CI 1.25-4.08), which when compared with the 3 weekly sessions set in the HEP, corresponded to 68.1% (95% CI 41.7%-136.1%) and was higher than the target set at ≥50% (*P*=.046). Different technical issues arose during the telerehabilitation meetings. No major technical issue necessitating canceling a meeting occurred, but minor technical problems occurred in 11% (5/47) of meetings. Echo voices were present with 1 participant, and this was resolved with the muting option: when the therapists or the participant spoke, the others muted themselves. The therapist lost the connection in one meeting for a few seconds and had to reconnect. On one occasion, a participant had difficulty with the sound output on their computer and had to join the meeting with their phone as an alternative. Regarding issues with the activity monitor, 5 participants had difficulties syncing the device, and the project coordinator resolved this problem by sharing the screen during the telerehabilitation meetings to review the required steps for setting it up.

All participants completed the pre- and postintervention questionnaires, except for 1 participant who did not return the data log sheet and never synced their activity monitor; therefore, they were excluded from the HEP compliance calculation despite reporting having performed their HEP regularly. For the ROM measurements, the missing values for each joint can be found in Multimedia Appendix 2. All 7 participants reported being comfortable communicating through the telerehabilitation platform, and the clarity of the video and audio of the platform was acceptable. The participants felt supported during the 12-week program, reported reduced travel time compared with if they had to visit the SHC in person, and expressed interest in using telerehabilitation in the future. All were satisfied or very satisfied with the remote evaluation, delivery of the HEP, follow-ups, and the overall organization of the 12-week program. The participants suggested increasing the span of the program (n=1), to include an in-person follow-up visit halfway through the program (n=1), and to include outcome measures targeting the adolescent age group (n=1). A summary of the improvements reported by the participants in the open-ended questions can be found in Table 1.

**Table 1:** Summary of the improvements reported in the open-ended questions (n=7).

Improvements reported	Participants, n (%)
Daily activities (transfers, descending stairs, walking ability, and greater efficiency propelling a manual wheelchair)	3 (43)
Strength	3 (43)
Endurance	2 (29)
Mobility	1 (14)
Balance	1 (14)

### Interrater Reliability

The interrater reliability varied among different joints, with an ICC of 0.985 (95% CI 0.980-0.989) for all joints combined. The lowest ICC was in forearm pronation with a median ICC of 0.252 (95% CI −0.477 to 0.75), and the highest was in shoulder extension with a median ICC of 0.998 (95% CI 0.983-1.00). The specific ICC for each joint is presented in Table 2.

**Table 2:** Intraclass correlation coefficient for each joint.

Joint	Number^a^	Median (95% CI)
Shoulder abduction	10	0.668 (0.072 to 0.908)
Shoulder flexion	4	0.915 (0.158 to 0.994)
Shoulder extension	5	0.998 (0.983 to 1)
Elbow flexion	12	0.988 (0.956 to 0.997)
Elbow extension	12	0.99 (0.965 to 0.997)
Forearm pronation	10	0.252 (−0.477 to 0.75)
Forearm supination	10	0.691 (0.069 to 0.917)
Wrist flexion	12	0.707 (0.277 to 0.904)
Wrist extension	12	0.858 (0.073 to 0.968)
Hip flexion	14	0.72 (0.315 to 0.901)
Hip extension	11	0.833 (0.484 to 0.952)
Hip internal rotation	11	0.975 (0.914 to 0.993)
Hip external rotation	12	0.971 (0.901 to 0.991)
Knee flexion	14	0.992 (0.958 to 0.998)
Knee extension	16	0.986 (0.961 to 0.995)
Ankle dorsiflexion	12	0.917 (0.749 to 0.975)
Ankle plantarflexion	12	0.878 (0.636 to 0.963)

^a^Number of data included for analysis, with a possible maximum of 16 (4 participants×2 sides×2 times).

### Effectiveness

For the GAS, the objectives varied among the participants and were related to pain management (n=2); endurance in manual labor (n=1), writing (n=1), standing (n=1), or walking (n=2); sports (sledge hockey: n=1; karate: n=1); and daily activities (buttoning up shirt: n=1; transfer ability: n=2; controlling stair descent: n=1; self-propelling a wheelchair: n=1; self-feeding with utensils: n=1). The median number of goals established with the participants was 2 (range 1-3). A total of 15 goals were established, and 12 goals were achieved. The 3 goals that were not achieved included pain management (n=2) and walking endurance (n=1). The overall program *t* score was 74.85, whereas the median *t* score among participants was 56.21 (95% CI 30-72.82); both scores are higher than the threshold of 50 points, meaning that overall, the participants achieved their goals.

The pre- and postintervention results from the different questionnaires are shown in Table 3. A statistically significant change was observed in the pain and comfort domain of the PODCI (*P*=.08). The number of participants with clinically important changes was as follows: upper extremity (improvement: n=2; decrease: n=1), transfers and mobility (improvement: n=3; decrease: n=2), sports and physical function (improvement: n=1; decrease: n=1), pain and comfort (improvement: n=1; decrease: n=0), happiness (improvement: n=2; decrease: n=1), and global function (improvement: n=2; decrease: n=0). For the PAQ-A, a statistically significant improvement of 14% was observed in the group (*P*=.046). Multimedia Appendices 3-4 show the pre- and postintervention results for each participant for the global domain of the PODCI and PAQ-A, respectively. For the APPT, 4 of 7 participants reported pain at baseline. One participant reported clinically meaningful improvement in the APPT at the end of the 12-week HEP; no changes were statistically significant.

For ROM, improvements in shoulder abduction (*P*=.08), shoulder flexion (*P*=.07), wrist extension (*P*=.05), and knee extension (*P*=.04) were found to be statistically significant. No significant changes were observed in the other joints. The results for ROM for each joint are shown in Table 4.

**Table 3:** Pre- and postintervention results from the different questionnaires.

Questionnaires		Preintervention, median (95% CI)	Postintervention, median (95% CI)	*P* value
**PODCI^a^**				
	Upper extremity	87.50 (12.50 to 100)	95.83 (4.17 to 100)	.79
	Transfer and mobility	93.94 (21.97 to 100)	91.66 (12.12 to 100)	.60
	Sports and physical function	79.86 (11.36 to 86.11)	63.64 (13.64 to 93.18)	.61
	Pain and comfort	71.11 (34.44 to 100)	85.00 (49.44 to 100)	*.08^b^*
	Happiness	85.00 (50.00 to 100)	90.00 (35.00 to 100)	.50
	Global function	87.47 (20.07 to 94.86)	82.08 (19.84 to 94.55)	.61
PAQ-A^c^		1.62 (1.00 to 2.82)	2.32 (1.00 to 3.45)	*.046*
**APPT^d^**				
	Location (number)	1 (0 to 6)	1 (0 to 6)	>.99
	Scale (cm)	1.55 (0 to 5.50)	1.10 (0 to 6.20)	.72
	Sensory (%)	5.40 (0 to 10.8)	2.70 (0 to 10.8)	>.99
	Affective (%)	0 (0 to 0)	0 (0 to 0)	>.99
	Evaluative (%)	0 (0 to 25.00)	0 (0 to 37.5)	>.99

^a^PODCI: Pediatric Outcomes Data Collection Instrument.

^b^Italicized values indicate a significance level of *P*<.10.

^c^PAQ-A: Physical Activity Questionnaire for Adolescents.

^d^APPT: Adolescent Pediatric Pain Tool.

**Table 4:** Range of motion.

Joint	Data included for analysis^a^	Preintervention (in degrees), median (95% CI)	Postintervention (in degrees), median (95% CI)	*P* value
Shoulder abduction	12	143 (16 to 159)	151.5 (25 to 163)	*.08^b^*
Shoulder flexion	6	34.5 (0 to 152)	57.5 (0 to 153)	*.07*
Shoulder extension	3	66 (66 to 75)	60 (50 to 66)	.11
Elbow flexion	14	146.5 (86 to 164)	144.5 (92 to 158)	.67
Elbow extension	11	−9 (−82 to 0)^c^	−4 (−95 to 9)^c^	.35
Forearm pronation	9	82 (65 to 91)	95 (78 to 102)	.12
Forearm supination	9	71 (44 to 86)	66 (49 to 84)	.48
Wrist flexion	8	86.5 (29 to 97)	78 (11 to 94)	.11
Wrist extension	8	20.5 (−20 to 50)	30 (−7 to 57)	*.05*
Hip flexion	12	109.5 (95 to 127)	108.5 (94 to 130)	.92
Hip extension	8	1.5 (−50 to 30)	−1.5 (−45 to 16)^c^	.25
Hip internal rotation	3	1 (1 to 27)	12 (10 to 27)	.18
Hip external rotation	4	30 (4 to 55)	33 (11 to 48)	.72
Knee flexion	12	105.5 (50 to 130)	115 (93 to 138)	.61
Knee extension	14	−18 (−63 to −7)^c^	−14.5 (−62 to −4)^c^	*.04*
Ankle dorsiflexion	12	−21 (−28 to 5)^c^	−20.5 (−29 to −2)^c^	.72
Ankle plantarflexion	12	32 (23 to 40)	29.5 (21 to 41)	.53

^a^Number of data included for analysis, with a possible maximum of 14 (7 participants×2 sides).

^b^Italicized values indicate a significance level of *P*<.10.

^c^Negative values represent a lack of range of motion. For example, a negative knee extension signifies an inability to achieve full extension. Range of motion was measured to whole degrees; 0.5° resulted from median calculations of even numbers.

## Discussion

### Principal Findings

In this study on the use of telerehabilitation to deliver an HEP to youth with AMC, it was found that this approach is feasible and well accepted by participants. The GAS was a feasible measure in this pilot study because it provided the individualization of goals for the youths living with AMC who presented with varying levels of joint involvement. Overall, most of the GAS goals were achieved at the end of the 12-week HEP. In addition, some clinically meaningful improvements were observed in the PODCI and APPT scores, showing promising effects of an HEP. We also explored the reproducibility of using a virtual goniometer in this study and found good-to-excellent agreement overall.

### Feasibility

Feasibility was demonstrated with the achievement of all operationalization criteria, except for the recruitment rates. The observed completion rate of 63.6% corresponded to the criteria set at ≥50%. One withdrawal was due to the need for more support from the caregiver to perform the HEP. Support from families or the entourage of the participant was important for most of the participants, specifically for the youngest participants and those with more severe joint involvement. The intervention in this study was semisupervised, with follow-ups provided every 3 weeks. The compliance of 68.1% (2.04/3 times per week) to the HEP measured in this study is closer to the compliance rate of 76% observed in supervised interventions [29] than to that of nonsupervised interventions (11%-37%) [30]. A total of 11% (5/47) of the telerehabilitation meetings were canceled on the same day, which is similar to the 12% (997/8306) observed for in-person appointments in the rehabilitation department at the SHC–Canada (unpublished data, 2019). Extreme weather in Canada often results in cancellation of appointments, but none were canceled for this reason in this study owing to its web-based care delivery. A few technical issues occurred during this study, but all were resolved promptly, and none affected the safety of the participants. Therefore, technical issues should not be a reason to preclude the use of telerehabilitation in future studies or in clinical practice.

Although the reproducibility of using a virtual goniometer varied across joints, this method of measuring ROM was nevertheless useful for setting individualized goals, developing the HEP, and performing the initial and final assessments. Some challenges with web-based ROM measurements included different movement planes for measurement, varying positions in which ROM measurements were taken according to the participants’ contractures (eg, elbow flexion in sitting or standing position), space restrictions in the home, or poor or high luminosity. In future studies using web-based ROM measurements, we recommend the use of guidelines for proper positioning, therapist training, contrasting clothing for the participants, proper luminosity, and laptop or tablet use to allow adjustment of the camera as needed.

### Effectiveness

The results regarding the intervention’s effectiveness are promising. The GAS score, which was the main outcome measure to explore effectiveness, showed significant improvement at the end of the 12-week HEP. Among the 12 goals achieved measured using the GAS, 9 were achieved beyond expectations: endurance in manual labor, standing, and walking; sports (karate); and daily activities (buttoning up shirt, transfer ability, controlling stair descent, self-propelling a wheelchair, and using a fork). These large improvements might be due to the scaling of the goals using the GAS table. As most of the participants did not perform exercises before participating in this project, they may have demonstrated quicker gains. However, 2 participants with chronic pain did not achieve their pain goal, perhaps because of the multifactorial etiology of pain requiring management by an interdisciplinary team [31]. Another participant did not meet their initial goal for walking endurance as per the GAS score because of overestimation of baseline levels as reported by the participant; nonetheless, improvements during the final assessment (quicker walking pace) were noted. As the GAS allows for individualized and varied goals, it proved to be a versatile and sensitive measurement tool, given the high level of variability of joint involvement and physical function. There are benefits to using the GAS, but this tool requires some level of training and experience to develop goals that are SMART (specific, measurable, achievable, realistic and timed) [32]. After determining individualized goals in collaboration with participants, therapists need to scale the goals according to an expected level of change and predict potential improvement within the timeline, which may not always be intuitive.

Although some clinically significant changes were measured with the PODCI, floor and ceiling effects were observed in a few participants. For example, in a participant with severe joint involvement, a floor effect was noted in all but the happiness domains. Large improvements in the transfer ability from wheelchair to exercise table in this participant were noted during the telerehabilitation sessions, yet the PODCI did not detect this change because the focus of the PODCI is mainly on ambulatory activities such as walking and running. The opposite effect occurred in participants who achieved maximum scores at baseline and, therefore, no positive change was detected. For these reasons, we conclude that the PODCI may not be an appropriate measure for our study, which used an exercise intervention for a heterogeneous group of youths. The baseline results from the PAQ-A were very low, yet they increased at the end of the 12-week HEP, with levels approaching those of typically developing adolescents [33]. The level of physical activity among participants with AMC remained lower than that among typically developing adolescents. This was similar to a study of adolescents with cerebral palsy [34]. With regard to ROM, we did not expect any significant changes because no participant selected ROM as their goal. Some statistically significant improvements were found, and they may have been explained by an improvement in muscle strength; however, these changes may not translate into clinically important changes.

### Limitations

Most of the outcomes were standardized, although the HEP was based on individualized goals. The assessment provided did not always correspond to the needs of the participants because they did not provide objective information related to the individualized goals (eg, strength or endurance). For example, joint-specific ROM was measured for all participants, but it was only pertinent for 1 participant who had a dressing goal. For the interrater reliability of ROM measurement with a virtual goniometer, it would be best to compare those values with in-person measurements. However, in the context of this study, it was not possible to do so because the study design entailed remote visits only. Therefore, the ROM results should be interpreted with caution. The selected questionnaires for this study were validated for children aged up to 18 years, which corresponded to most of the participants included in this study (5/7, 71%). However, at the SHC–Canada, patients are followed until 21 years of age, and an older participant mentioned that the content of some of the questionnaires was not adapted to their reality. Finding standardized measures devised and validated for both pediatric and young adult populations is a challenge because measures are typically developed for either pediatric or adult populations. As this was a pilot study with the main objective being the assessment of its feasibility, no control group was included. A larger multicenter randomized controlled trial would be needed to ascertain the study power needed to draw conclusions on the effectiveness of an HEP. Initial and final assessments performed in person with follow-ups conducted remotely could also be a good compromise for future studies.

### Conclusions

The results of this pilot study suggest that it is feasible to use telerehabilitation to deliver an HEP for youth with AMC having different functional levels, having various goals, and living across different geographical regions. This approach also holds promise regarding the effectiveness of an HEP. Lessons can be learned from the challenges and positive aspects denoted in this study, which could lead to enhanced future protocols.

## Appendix

Multimedia Appendix 1 An exercise program is provided as an example for each participant.

Multimedia Appendix 2 This table shows the missing values and their associated reasons for each range of motion measurement.

Multimedia Appendix 3 A bar graph that shows the pre- and postintervention global function scores of the Pediatric Outcomes Data Collection Instrument for each participant and the clinically significant difference. *Medium clinically significant difference, ** Large clinically significant difference.

Multimedia Appendix 4 A bar graph that shows the pre- and postintervention Physical Activity Questionnaire for Adolescents results for each participant. *Difference between 10% and 19%; **difference between 20% and 29%; ***difference ≥30%.

## Abbreviations

AMC: arthrogryposis multiplex congenita
APPT: Adolescent and Pediatric Pain Tool
GAS: Goal Attainment Scale
HEP: home exercise program
ICC: intraclass correlation coefficient
PAQ-A: Physical Activity Questionnaire for Adolescents
PODCI: Pediatrics Outcomes Data Collection Instrument
PRT: physical rehabilitation therapist
ROM: range of motion
SHC: Shriners Hospitals for Children
SMART: specific, measurable, achievable, realistic, and timed

## References

[1] Lowry RB, Sibbald B, Bedard T, Hall JG (2010). Prevalence of multiple congenital contractures including arthrogryposis multiplex congenita in Alberta, Canada, and a strategy for classification and coding. Birth Defects Res A Clin Mol Teratol.

[2] Dahan-Oliel N, Cachecho S, Barnes D, Bedard T, Davison AM, Dieterich K, Donohoe M, Fąfara A, Hamdy R, Hjartarson HT, S Hoffman N, Kimber E, Komolkin I, Lester R, Pontén E, van Bosse HJ, Hall JG (2019). International multidisciplinary collaboration toward an annotated definition of arthrogryposis multiplex congenita. Am J Med Genet C Semin Med Genet.

[3] Hall J (2014). Arthrogryposis (multiple congenital contractures): diagnostic approach to etiology, classification, genetics, and general principles. Eur J Med Genet.

[4] Hall J, Kimber E, van Bosse HJ (2017). Genetics and classifications. J Pediatr Orthop.

[5] Hall J, Reed S, Driscoll E (1983). Part I. Amyoplasia: a common, sporadic condition with congenital contractures. Am J Med Genet.

[6] Staheli L, Hall J, Jaffe KM, Paholke DO (1999). Arthrogryposis: A Text Atlas.

[7] Dubousset J, Guillaumat M (2015). Long-term outcome for patients with arthrogryposis multiplex congenita. J Child Orthop.

[8] Dillon E, Bjornson K, Jaffe K, Hall J, Song K (2009). Ambulatory activity in youth with arthrogryposis: a cohort study. J Pediatr Orthop.

[9] Rodrigues E, Gomes A, Tanhoffer A, Leite N (2014). Effects of exercise on pain of musculoskeletal disorders: a systematic review. Acta Ortop Bras.

[10] Skalsky AJ, McDonald CM (2012). Prevention and management of limb contractures in neuromuscular diseases. Phys Med Rehabil Clin N Am.

[11] Kroksmark A, Kimber E, Jerre R, Beckung E, Tulinius M (2006). Muscle involvement and motor function in amyoplasia. Am J Med Genet A.

[12] Gagnon M, Caporuscio K, Veilleux LN, Hamdy R, Dahan-Oliel N (2019). Muscle and joint function in children living with arthrogryposis multiplex congenita: a scoping review. Am J Med Genet C Semin Med Genet.

[13] Elfassy C, Darsaklis V, Snider L, Gagnon C, Hamdy R, Dahan-Oliel N (2020). Rehabilitation needs of youth with arthrogryposis multiplex congenita: perspectives from key stakeholders. Disabil Rehabil.

[14] Wagner LV, Cherry JS, Sawatzky BJ, Fąfara A, Elfassy C, Eriksson M, Montpetit K, Bucci T, Donohoe M (2019). Rehabilitation across the lifespan for individuals with arthrogryposis. Am J Med Genet C Semin Med Genet.

[15] Moffet H, Tousignant M, Nadeau S, Mérette C, Boissy P, Corriveau H, Marquis F, Cabana F, Ranger P, Belzile EL, Dimentberg R (2015). In-home telerehabilitation compared with face-to-face rehabilitation after total knee arthroplasty: a noninferiority randomized controlled trial. J Bone Joint Surg Am.

[16] Tousignant M, Moffet H, Nadeau S, Mérette C, Boissy P, Corriveau H, Marquis F, Cabana F, Ranger P, Belzile EL, Dimentberg R (2015). Cost analysis of in-home telerehabilitation for post-knee arthroplasty. J Med Internet Res.

[17] Keshet D, Bernstein M, Dahan-Oliel N, Ouellet J, Pauyo T, Rabau O, Saran N, Hamdy R (2020). Management of common elective paediatric orthopaedic conditions during the COVID-19 pandemic: the Montreal experience. J Child Orthop.

[18] Camden C, Pratte G, Fallon F, Couture M, Berbari J, Tousignant M (2020). Diversity of practices in telerehabilitation for children with disabilities and effective intervention characteristics: results from a systematic review. Disabil Rehabil.

[19] Gagnon M, Collins J, Elfassy C, Merlo GM, Marsh J, Sawatzky B, Yap R, Hamdy R, Veilleux LN, Dahan-Oliel N (2020). A telerehabilitation intervention for youths with arthrogryposis multiplex congenita: protocol for a pilot study. JMIR Res Protoc.

[20] Durfee WK, Savard L, Weinstein S (2007). Technical feasibility of teleassessments for rehabilitation. IEEE Trans Neural Syst Rehabil Eng.

[21] Koo TK, Li MY (2016). A guideline of selecting and reporting intraclass correlation coefficients for reliability research. J Chiropr Med.

[22] McGraw KO, Wong SP (1996). Forming inferences about some intraclass correlation coefficients. Psychol Methods.

[23] Shrout P, Fleiss J (1979). Intraclass correlations: uses in assessing rater reliability. Psychol Bull.

[24] Bogaerts J, Sydes MR, Keat N, McConnell A, Benson A, Ho A, Roth A, Fortpied C, Eng C, Peckitt C, Coens C, Pettaway C, Arnold D, Hall E, Marshall E, Sclafani F, Hatcher H, Earl H, Ray-Coquard I, Paul J, Blay J, Whelan J, Panageas K, Wheatley K, Harrington K, Licitra L, Billingham L, Hensley M, McCabe M, Patel PM, Carvajal R, Wilson R, Glynne-Jones R, McWilliams R, Leyvraz S, Rao S, Nicholson S, Filiaci V, Negrouk A, Lacombe D, Dupont E, Pauporté I, Welch JJ, Law K, Trimble T, Seymour M (2015). Clinical trial designs for rare diseases: studies developed and discussed by the International Rare Cancers Initiative. Eur J Cancer.

[25] Kiresuk T, Smith A, Cardillo J (2014). Goal Attainment Scaling: Applications, Theory, and Measurement.

[26] Oeffinger D, Bagley A, Rogers S, Gorton G, Kryscio R, Abel M, Damiano D, Barnes D, Tylkowski C (2008). Outcome tools used for ambulatory children with cerebral palsy: responsiveness and minimum clinically important differences. Dev Med Child Neurol.

[27] Farrar J, Young JJ, LaMoreaux L, Werth J, Poole M (2001). Clinical importance of changes in chronic pain intensity measured on an 11-point numerical pain rating scale. Pain.

[28] Tsze DS, Hirschfeld G, von Baeyer CL, Suarez LE, Dayan PS (2019). Changes in pain score associated with clinically meaningful outcomes in children with acute pain. Acad Emerg Med.

[29] Wong YK, Hui E, Woo J (2005). A community-based exercise programme for older persons with knee pain using telemedicine. J Telemed Telecare.

[30] Hartman A, te Winkel M, van Beek R, de Muinck S, Kemper H, Hop W, van den Heuvel-Eibrink M, Pieters R (2009). A randomized trial investigating an exercise program to prevent reduction of bone mineral density and impairment of motor performance during treatment for childhood acute lymphoblastic leukemia. Pediatr Blood Cancer.

[31] Manchikanti L, Singh V, Kaye A, Hirsch J (2020). Lessons for better pain management in the future: learning from the past. Pain Ther.

[32] Bovend'Eerdt T, Botell R, Wade D (2009). Writing SMART rehabilitation goals and achieving goal attainment scaling: a practical guide. Clin Rehabil.

[33] Fernández I, Canet O, Giné-Garriga M (2017). Assessment of physical activity levels, fitness and perceived barriers to physical activity practice in adolescents: cross-sectional study. Eur J Pediatr.

[34] Maher C, Williams M, Olds T, Lane A (2007). Physical and sedentary activity in adolescents with cerebral palsy. Dev Med Child Neurol.

