# Supplementary material for: Using Telerehabilitation to Deliver a Home Exercise Program to Youth With Arthrogryposis: Single Cohort Pilot Study
Source: J Med Internet Res. 2021 Jul 6;23(7):e27064. doi: 10.2196/27064 (PMC8292936; doi:10.2196/27064)
Supplement: Multimedia Appendix 1 [file jmir_v23i7e27064_app1.pdf]

Shriners Hospitals for Children
1003, boulevard Décarie
Montreal, QC H4A 0A9
514-282-7189
hopitauxshrinerspourenfants.org/Canada

Programme d'exercices #1

Goals:
1. Self-feed with less compensatory techniques
2. Self-propelling wheelchair
3. Ability to transfer in a car

Notes : Repos 1-2 minutes entre chaque série.

1 Flexion coude isométrique

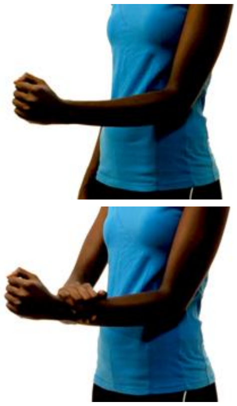

Séries: 2-3 Réps: 10 Tenir: 5 secondes

Placez la main sur le poignet du côté atteint.
Tentez de plier le coude tout en empêchant le mouvement avec votre main.
Il ne devrait pas avoir plus de mouvement.
Assurez-vous de forcer au maximum.

2 Extension iso. du coude

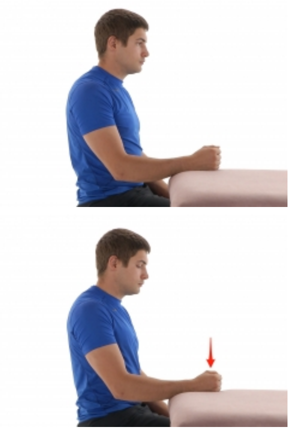

Séries: 2-3 Réps: 5 secondes

Assoyez-vous bien droit devant une table.
Formez un poing avec votre main et placez le poing sur la table, le coude fléchi à environ 90 degrés.
Essayez de déplier le coude en poussant vers le bas contre la table.
Maintenez la contraction pour la durée recommandée, relâchez et répétez.

3 Flexion de hanche, assis

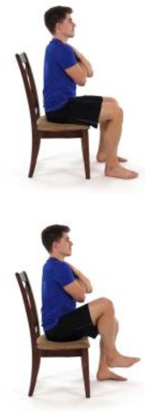

Séries: 3 Réps: 10 Poids: avec assistance de tes propres main; la descente est la phase la plus importante Tempo: 2:3

Assoyez-vous sur une chaise droite avec le dos en position neutre (légèrement arqué).
En gardant le genou fléchi, levez le genou vers la poitrine en ne roulant pas le bassin vers l'arrière quand vous effectuez le mouvement.
Abaissez et répétez.

© Physiotec 1996-2019. Tous droits réservés

Shriners Hospitals for Children
1003, boulevard Décarie
Montreal, QC H4A 0A9
514-282-7189
hopitauxshrinerspourenfants.org/Canada

4 Flex./ext. coude assis

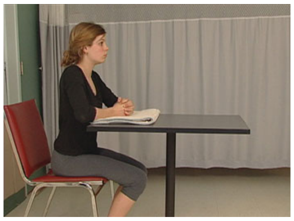
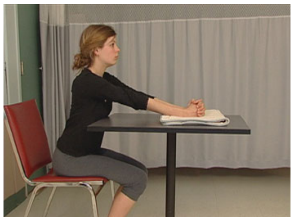

Séries: 3 Réps: 10 - cette exercice est soit fait avec maman ou avec serviette pour diminuer la friction; idéalement, l'épaule est au meme niveau que le coude

Assoyez-vous.
Appuyez les bras sur la table, les coudes pliés et placez une serviette sous vos mains.
Allongez le bras en glissant la main sur la table.
Poursuivez le mouvement en poussant les épaules vers l'avant.
Revenez à la position de départ en pliant les coudes.

---

5 Extension coude serviette

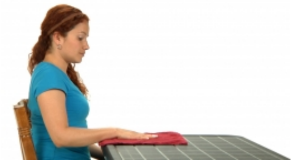
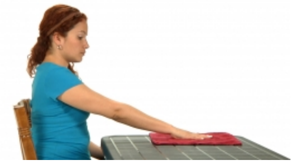

Séries: 3 Réps: 10 Poids: avec assistance de tes propres main; la descente est la phase la plus importante Tempo: 2:3

Assoyez-vous.
Appuyez un avant-bras sur la table et placez une serviette sous votre main.
Gardez le coude plié.
Allongez le bras en glissant la main sur la table.
Poursuivez le mouvement jusqu'à ce que le coude soit allongé complètement.
Revenez à la position de départ en pliant le coude.

Attention à ne pas forcer avec votre tronc et votre nuque.

© Physiotec 1996-2019. Tous droits réservés

Shriners Hospitals for Children
1003, boulevard Décarie
Montreal, QC H4A 0A9
514-282-7189
hopitauxshrinerspourenfants.org/Canada

Programme d'exercices #1

6 Extension hanche sur chaise

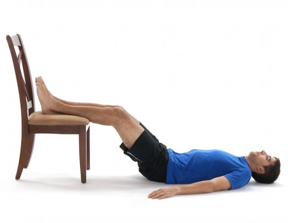

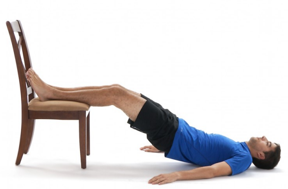

Séries: 3 Réps: 10 Poids: avec assistance de tes propres main; la descente est la phase la plus importante Tempo: 2:3

Couchez-vous sur le dos et placez vos jambes sur la chaise.
Décollez le bassin du sol pour faire le pont en serrant les fesses.
Descendez sur le sol et répétez.

© Physiotec 1996-2019. Tous droits réservés

Shriners Hospitals for Children
1003, boulevard Décarie
Montreal, QC H4A 0A9
514-282-7189
hopitauxshrinerspourenfants.org/Canada

Home Exercise Program #1

Goals:
1. Ability to get in a car
2. Control stair decent
3. Kick higher in karate

Notes : - All exercises should be done on both legs.

1 Step-down

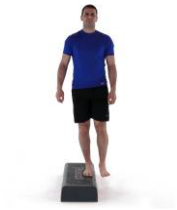

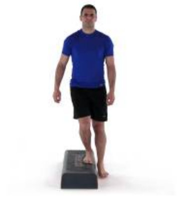

Sets: **3** **Reps:** **10** **Freq:** **daily** **Tempo:** **2 seconds up, 2 seconds down, SLOW** **Rest:** **1 minute rest in between sets**

Stand up straight on a stool supported on one leg. Make sure you have a counter top or railing that is safe and secure beside you for support. You will need to hold throughout.
Align your knee over your toes and slowly beginning to bend at the hips (sending the bum back) and then bending the knee as if you were to sit in a chair.
As you bend, the unsupported leg will lower slowly.
Do not pass your standing knee over your toes and make sure that your heel stays as flat as possible (on the stool).
You do not need to touch the ground; it is more of a mini single-leg squat.
Return to the starting position by unbending the knee of the leg on the stool.
Straighten the knee completely and stand upright before placing the free foot onto the stool.
Repeat.

© Physiotec 1996-2019. All rights reserved

Shriners Hospitals for Children
1003, boulevard Décarie
Montreal, QC H4A 0A9
514-282-7189
hopitauxshrinerspourenfants.org/Canada

2 Hip+knee auto-assisted flexion

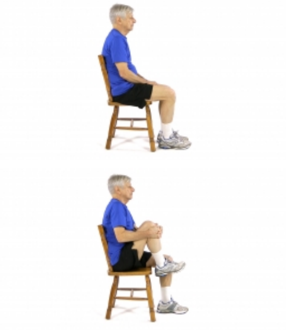

Sets: 3 Reps: 10 Freq: daily Rest: 1-2 minutes in between sets

Start in a seated position and surround one knee with your hands.
With the help of your hands, ACTIVELY bring your knee towards your chest and help yourself for the range that is difficult to accomplish.
As you lower your leg, ACTIVELY try to slow the descent as if you wanted to hold your leg in position while gradually releasing your hold.
Repeat.

You may also use a towel or belt looped under your thigh to assist.

---

3 Standing hip flexion w/ stool

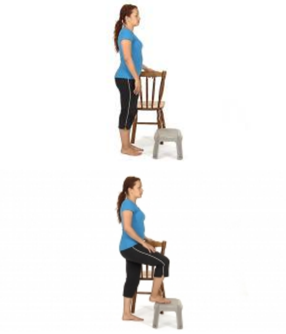

Sets: 2-4 Reps: 15-20 Freq: daily Rest: 1 minute in between sets

Stand up straight beside a chair with a stool in front of you.
Slowly lift your foot up onto the stool, step or textbook by bending your hip, knee, and ankle as much as you can.
Lower your foot to the ground by lifting up and off with control.
When you lift your leg, do not move the rest of your body and attempt not to knock the bench. Your abdominals should be engaged and you should be looking tall and far in front of you.
You can use the number of times you knock the object as a test to track your progress.

© Physiotec 1996-2019. All rights reserved

Shriners Hospital For Children
1003, boulevard Décarie
Montreal, QC
H4A 0A9
514-282-7189
hopitauxshrinerspourenfants.org/Canada

Goal:
To improve walking endurance

Notes:

1- Wall squat - Sets:2-3 / Repetition:5 / Frequency:3x per week at minimum / Hold:10 seconds / Rest:1.5 minute in between sets

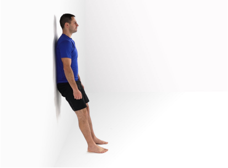

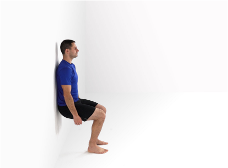

Stand up against a wall with your feet in front of you; when your thighs are parallel to the ground your knees should be at appropriately 90 degrees. Slide down the wall, keeping the upper body in contact with the wall. Keep the knees in line with the feet. Your knees should not pass your toes and your heels should remain flat on the ground. Do not use your hands. Slowly stand back up squeezing the buttocks and thigh muscles as you return to your starting position.
Repeat.

2- Bridging - Sets:3 / Repetition:10 / Frequency:3x per week at minimum / Hold:2 second hold while bum is in the air / tempo:2 seconds up / Rest:1 minute in between sets

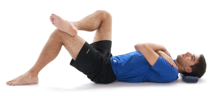

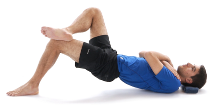

Lie on your back with your knees bent and one leg crossed over the other. Contract your abdominal muscles and your buttocks while lifting your buttocks off the ground.
Slowly return to the initial position and repeat.

Progressions possible (in appropriate order):
- place a small stool under supporting limb
- progress to perform with the other leg unsupported (that is actively held, fully extended, in the air)

© Physiotec 1996 - 2019. All rights reserved.

Shriners Hospital For Children
1003, boulevard Décarie
Montreal, QC
H4A 0A9
514-282-7189
hopitauxshrinerspourenfants.org/Canada

3- Clamshell - Sets:3 / Repetition:10 / Weight:use appropriate elastic so that you may complete full movement but last two or three repetitions of each set are difficult / Frequency:3x per week at minimum / tempo:2 seconds open / Rest:1 minute in between each set

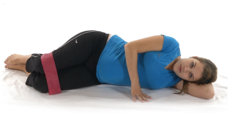

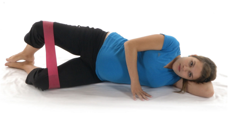

Lie on your side with your knees bent and head supported with a resistance band about 1 inch above your knees.
Lift your upper knee without moving your pelvis or lifting the foot off your other foot, in order to create a gap between your thighs.
Lower your knee and repeat.

Once completed, perform the same exercise with the opposite leg on top.

4- Isometric plantar flexion with band and towel (please ignore equipment in image; movement remains the same) - Sets:3 / Repetition:12 / Frequency:3x per week at minimum / Hold:5 second hold / Rest:1 minute rest in between sets

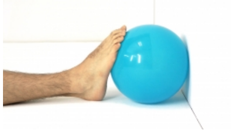

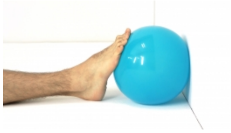

Two exercises to complete, one foot at a time:
1. Against tough elastic band with knee bent to 90 deg

2. Against towel with back supported against a wall and knee fully extended
- make sure that with this exercise is completed without allowing any motion in the ankle (you must match your resistance)

Both exercises involving initiating the movement of pointing your toes.

© Physiotec 1996 - 2019. All rights reserved.

Shriners Hospital For Children
1003, boulevard Décarie
Montreal, QC
H4A 0A9
514-282-7189
hopitauxshrinerspourenfants.org/Canada

Goals:

1. Improve walking endurance
2. Quadriceps strengthening/ reduce pain on thigh

Notes: Walking: Must walk 5 minutes straight and repeat this activity 3 times in one evening. TO BE DONE EVERY DAY.

All other exercises should be done 3x per week.

Recommendations include:
- to walk with braces in home as much as possible and use wheelchair as little as possible
- to walk in the community (ex. push wheelchair in grocery store, at the mall, in school corridors, etc.)
- wear watch at all times, if not, at minimum, when performing home exercise program

1- Bridge - Sets:2 / Repetition:10 / Weight:pillow betweens legs / Hold:5 second hold / tempo:2 seconds to lift bum / Rest:1.5 minutes in between each set

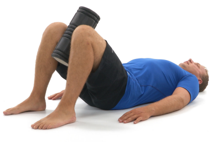

Lie down on your back with your knees bent and a pillow between them. Lift your bum off the ground. As you lift, squeeze the pillow between your legs.
Hold for a few seconds on top then slowly lower your bum.
Repeat.

Make sure you breathe out every time you lift your bum up.

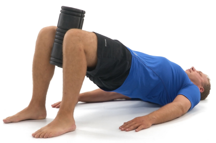

2- Leg lift - Sets:2 / Repetition:10 / Hold:2 seconds with the leg in the air / tempo:slow on the way up and down / Rest:1.5 minutes in between sets

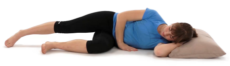

Lay on your side with your head supported and lower leg bent. Make sure the other hand is holding the ground for stability.
Lift the top leg up as the picture shows, keeping the toes pointing forward and the leg aligned with the body. Make sure you are not rolling forward or backward.
Lower the top leg and repeat.

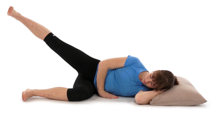

© Physiotec 1996 - 2019. All rights reserved.

Shriners Hospital For Children
1003, boulevard Décarie
Montreal, QC
H4A 0A9
514-282-7189
hopitauxshrinerspourenfants.org/Canada

3- Sit to stand using STOOL - Sets:2 / Repetition:8 / Rest:2 minutes of rest in between sets

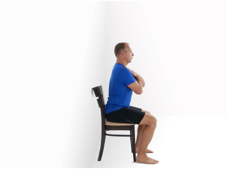

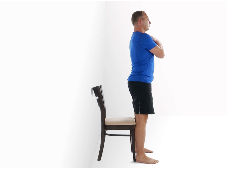

Sit on the stool (placed against a wall to prevent it from moving).
Fold your arms across your chest or simply avoid using your hands.
With your feet slightly apart, lean forward and stand up.
Do not bend your back or jump up.
Slowly sit back down.
Repeat.

© Physiotec 1996 - 2019. All rights reserved.

Shriners Hospital For Children
1003, boulevard Décarie
Montreal, QC
H4A 0A9
514-282-7189
hopitauxshrinerspourenfants.org/Canada

Goals:

1. To use more the left arm during sledge hockey
2. Buttoning up shirt

Notes: To be done: 3x per week at minimum

1- Wall slide + lean into it - Sets:2 / Repetition:10 / Hold:5 seconds

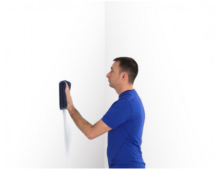

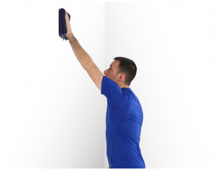

Stand up facing to a wall and place your hand on the wall, with a towel between the wall and your hand.
Slide up as far as you can.
Let yourself lean in towards the wall to feel a stretch under the arm pit area.
Slide your hand back down.
Repeat the exercise.

2- Shoulder flexion with elastic - Sets:3 / Repetition:10 / Weight:red elastic / tempo:4-5 seconds up / Rest:1 mintute in between sets

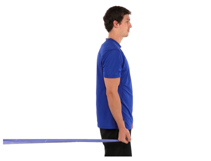

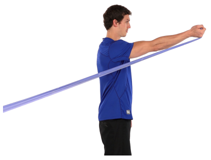

Tie an elastic band at hip height to a secure object behind you (door knob of closed door) or chair with someone sitting on it.
Start by holding the elastic band with your arm by your side. Do not follow the video for hand placement; it is preferred that you keep a thumbs up position by making a fist around the elastic (do not loop it around your hand or wrist).
Focus on keeping your arm locked the entire time (do not let your wrist fall or your elbow bend).
Stand as far forward as you can so that you may feel a small stretch in the front of your shoulder.
Raise your arm in front of you to nose or eye level.
Slowly return to the starting position.

3- Assisted shoulder extension with hockey stick - Sets:2 / Repetition:10 / Hold:5 seconds

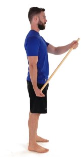

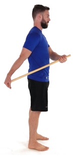

Stand straight with your chin tucked in holding a stick.
Place your left arm along your side and the other arm straight in front.
Push on the stick with your right arm to push the left shoulder back as high as you can, without poking your neck forward or causing pain.
Maintain the position, lower your arm and repeat.

© Physiotec 1996 - 2019. All rights reserved.

Shriners Hospital For Children
1003, boulevard Décarie
Montreal, QC
H4A 0A9
514-282-7189
hopitauxshrinerspourenfants.org/Canada

4- Grip strength with ball - Sets:3 / Repetition:10 / Hold:3 seconds / Rest:1 minute in between sets

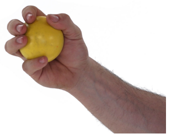

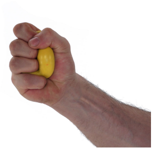

Remember to have your forearm supported by the table in front of you.
The side of your hand with your little finger should be resting on the table.
Hold a small ball in your hand and squeeze it as hard as you can.
Hold 3 seconds.
Release the ball slowly.
Repeat.

5- Full grip with putty - Weight:once putty received; fluorescent red

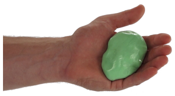

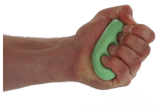

Make a fist with your fingers and thumb as you squeeze the putty. Make sure your forearm is supported on the table and that you are keeping your wrist as close to a 'thumbs up' position as possible.
Dig all the fingers in.
Hold 3 seconds.
Release grip.
Reshape the putty, if necessary, and repeat.

6- Standing plantar flexion - Sets:3 / Repetition:15 / tempo:2 seconds up

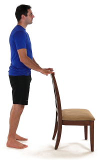

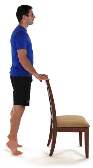

Stand on both feet on the floor next to a chair or table (for balance purposes).
Come onto the toes (raise heels) of both feet without bending the knee.
Keep the bum, tummy and thighs tucked tight.
Slowly bring your heels back down and repeat.

Rest between 1-2 minutes in between each set.

© Physiotec 1996 - 2019. All rights reserved.

Goal: Pain management

Shriners Hospital For Children
1003, boulevard Décarie
Montreal, QC
H4A 0A9
514-282-7189
hopitauxshrinerspourenfants.org/Canada

Notes: Recommendations:

- be conscious of good sitting posture (steps to take: tuck in tummy by drawing belly button in without cutting off your breath, bum shift to left and align shoulders with hips)
- continue regular physiotherapy exercises (emphasis on hip flexor stretching and adductor range of motion)
- remember to exhale during strenuous part of each exercise

1- Seated hip flexion - Sets:2 / Repetition:10 each leg / Frequency:2-3x per wk / tempo:2 seconds up / Rest:2 minute rest in between sets

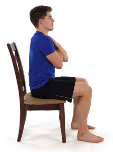

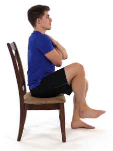

Sit on a straight chair with your back in neutral position.
Keeping your knee bent, lift your thigh off the chair as high as you can. Do not roll your pelvis backwards or lean back into your chair as you lift.
Lower and repeat.

Assistance required: helper
1. minimal on L
2. moderate on R

2- Hip adductor strengthening - Sets:3 / Repetition:5 / Frequency:2-3x per week / Hold:5 seconds / Rest:2 minute rest in between sets

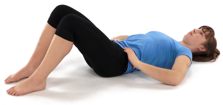

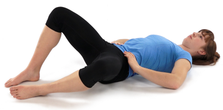

*** PLEASE IGNORE VIDEO. FOLLOW INSTRUCTIONS AND REFER TO EXERCISE POSITION.

Lie onto your back.
Have someone place your knees and hips in a bent position so that your feet are practically flat on the floor. (crook-lying position)
Engage your core by contracting your abdominal muscles.
Keep your legs in this position, with your knees pointing up at the ceiling, squeezing the inside of your thighs together x 5 seconds.
Slowly let your legs fall into the hands of your helper.
Repeat.

© Physiotec 1996 - 2019. All rights reserved.

Shriners Hospital For Children
1003, boulevard Décarie
Montreal, QC
H4A 0A9
514-282-7189
hopitauxshrinerspourenfants.org/Canada

3- Clamshell - Sets:2 / Repetition:10 each side / Frequency:2-3x per wk / tempo:2 seconds up / Rest:2 minute rest in between sets

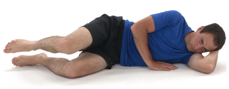

Lie on your side with your knees bent and head supported.
Elevate the top leg slightly so it doesn't touch the bottom leg.
Keep your top foot directly above the bottom one and lift/rotate your top knee without rolling back.
Lower your knee and repeat.
Breathe normally during this exercise.

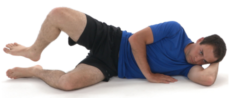

4- Hip extension strengthening - Sets:3 / Repetition:5 each leg / Frequency:2-3x per wk / tempo:2 seconds up / Rest:2 minutes in between sets

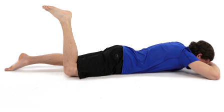

Lay with the face down and the affected side knee bent at 90 °. Your helper should help ensure the best alignment possible, meaning thigh brought in and foot up towards the ceiling as displayed. The best way to accomplish this is by placing one hand under the front of thigh and the other hand holding the lower leg, just above the ankle .
Lift the thigh off the surface.
Slowly lower to the start position.
Repeat the exercise.

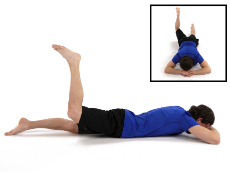

5- Passive hip internal rotation stretch - Sets:3 / Frequency:2-3x per wk / Hold:30 seconds / Rest:30 seconds-1 minute in between

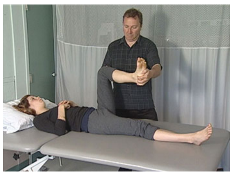

Place one hand under the knee and cup the heel with your other hand.
Using both hands, bend the hip and knee up until the thigh is perpendicular to the body and the shin is perpendicular to the thigh, all the while keeping the knee in midline.
Move one hand to the outside of the knee and the other to the inside of the heel and rotate the lower leg as far as possible towards the OUTSIDE without letting the knee fall in towards the patient's midline.
Hold x 30 seconds.
Come back to center and release.

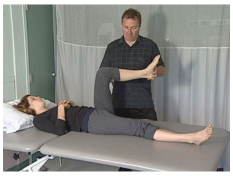

* NO NEED TO PERFORM HIP EXTERNAL ROTATION STRETCH IN OTHER DIRECTION (foot towards inside) AS DEPICTED IN THE VIDEO.

© Physiotec 1996 - 2019. All rights reserved.

Shriners Hospital For Children
1003, boulevard Décarie
Montreal, QC
H4A 0A9
514-282-7189
hopitauxshrinerspourenfants.org/Canada

Objectives:
1. Manual dexterity
2. Hand endurance
3. Standing endurance

Notes: Recommendations:

- si l'élastique ou pâte à modeler utilisé devient facile, vous pouvez progresser la résistance d'un niveau (putty rose fluo à vert, élastique facile à moyen à difficile)
- à faire programme d'exercice 2-3x par semaine pour débuter

N'hésitez pas si vous avez des questions!

1- Montée/descente de marche - Séries:2-3 / Répétition:10 / tempo:2 sec. montée / Repos:1 minute entre séries du même côté

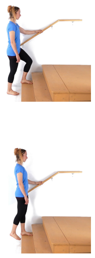

Tenez-vous sur une marche avec la jambe à renforcer.
Placez la majorité du poids du corps sur le talon de la jambe d'appui.
Inclinez votre poids sur la jambe sur la marche.
Montez sur la marche lentement, tout en gardant le genou aligné vers l'avant (non l'extérieur ou l'intérieur) et les hanches au niveau. Gardez le dos droit; évitez de pencher le tronc.
Descendez en ramenant la jambe libre vers le sol.

Répétez avec la jambe opposé. Suggérons de tenir avec minimum une main pour débuter et favoriser un bon contrôle du mouvement.

2- Montée de marche latérale - Séries:2-3 / Répétition:10 / Tenir:2 sec. montée / Repos:1 min. entre séries du meême côté

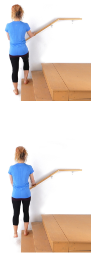

Tenez-vous de côté à un escalier. Suggérons de tenir avec une main au minimum pour favoriser un bon contrôle excentrique (phase descendante).
Montez la marche avec la jambe du coté de la marche, en gardant le genou aligné vers l'avant et les hanches au niveau. Utilisez le talon de la jambe d'appui pour monter plutôt que de pousser avec la jambe libre.
Descendez en ramenant la jambe saine vers le sol.

JAMBE DROITE:
- utilisez livres solides, placé de manière sécuritaire, proche d'une rampe ou un meuble ou chaise solide

JAMBE GAUCHE:
- utilisez l'escalier standard

3- Pas latéraux avec élastique - Séries:4 / Répétition:10 pas allez / Poids:élastique facile / tempo:2 secondes par pas / Repos:1-2 minutes

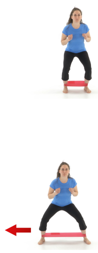

Placement de l'élastique au-dessus des genoux devrait être fait avec pieds collés.
Débutez en position de squat partielle avec un élastique autour des CUISSES (ne vous fier pas à l'image).
Effectuez des pas latéraux.
Gardez les genoux pointant vers l'avant et pieds écartés la largeur du bassin.
Chaque pas doit couvrir environ 50% de la distance initiale entre les deux pieds.

© Physiotec 1996 - 2019. Tous droits réservés

Shriners Hospital For Children
1003, boulevard Décarie
Montreal, QC
H4A 0A9
514-282-7189
hopitauxshrinerspourenfants.org/Canada

4- Opposition avec pâte - Séries:3 / Répétition:5

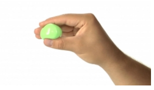
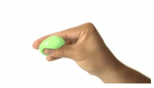

Opposez le pouce avec chaque doigt séparément et serrez la pâte entre les deux.
Répétez ou maintenez selon les directives.

À faire:
1. pouce + index
2. pouce + majeure
3. pouce + annulaire
4. pouce + auriculaire

5- Crochet des doigts - Séries:3 / Répétition:5

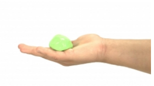
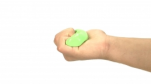

Faites un crochet avec les doigts en serrant la pâte.
Utilisez seulement les quatre doigts.
Répétez ou maintenez selon les directives.

6- Serrer le poing, pâte - Séries:3 / Répétition:10

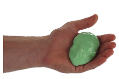
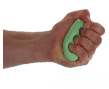

Serrez la pâte avec les doigts et le pouce.
Répétez ou maintenez selon les directives.

© Physiotec 1996 - 2019. Tous droits réservés

7- Adduction pouce, pâte - Séries:3 / Répétition:10

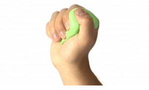
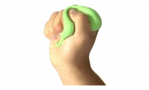

Placez la pâte dans la paume et fléchissez les doigts légèrement. Avec le pouce, poussez dans la pâte contre l'index.
Ne poussez pas sous l'index.
Remodelez la pâte et répétez.

8- Adduction doigts, pâte - Séries:2 / Répétition:5

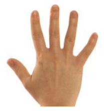
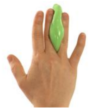

Serrez vos doigts ensemble sans les plier contre la pâte à modeler.

À faire:
1. index + majeure
2. majeure + annulaire
3. annulaire + auriculaire

9- Abduction des doigts avec pâte - Séries:3 / Répétition:8

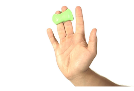
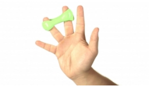

Placez la pâte autour d'une paire de doigt à la fois puis tentez de les écarter.
Remodelez la pâte et répétez.

À faire:
1. index + majeure
2. majeure + annulaire
3. pouce + index

© Physiotec 1996 - 2019. Tous droits réservés
