# Supplementary material for: Using Telerehabilitation to Deliver a Home Exercise Program to Youth With Arthrogryposis: Single Cohort Pilot Study
Source: J Med Internet Res. 2021 Jul 6;23(7):e27064. doi: 10.2196/27064 (PMC8292936; doi:10.2196/27064)
Supplement: Multimedia Appendix 2 [file jmir_v23i7e27064_app2.docx]

**Multimedia Appendix 2:** Missing values and their associated reasons for each range of motion measurement.

| **Column1** | **Not assessed** | **Assessed in the wrong plane** | **Not assessed with the same method** | **Total** |
| --- | --- | --- | --- | --- |
| Shoulder abduction | 2 | 0 | 0 | 2 |
| Shoulder adduction | 28 | 0 | 0 | 28 |
| Shoulder flexion | 0 | 12 | 0 | 12 |
| Shoulder extension | 17 | 0 | 0 | 17 |
| Elbow flexion | 0 | 0 | 0 | 0 |
| Elbow extension | 0 | 0 | 0 | 0 |
| Forearm pronation | 4 | 0 | 0 | 4 |
| Forearm supination | 2 | 0 | 0 | 2 |
| Wrist flexion | 0 | 0 | 2 | 2 |
| Wrist extension | 0 | 0 | 2 | 2 |
| Hip flexion | 0 | 0 | 2 | 2 |
| Hip extension | 6 | 1 | 0 | 7 |
| Hip internal rotation | 9 | 2 | 0 | 11 |
| Hip external rotation | 8 | 2 | 0 | 10 |
| Knee flexion | 0 | 0 | 2 | 2 |
| Knee extension | 0 | 0 | 0 | 0 |
| Ankle dorsiflexion | 4 | 0 | 0 | 4 |
| Ankle plantarflexion | 4 | 0 | 0 | 4 |
| **Total** | 84 | 17 | 8 | 109 |
