# Supplementary figures and images for: Using Telerehabilitation to Deliver a Home Exercise Program to Youth With Arthrogryposis: Single Cohort Pilot Study
Source: J Med Internet Res. 2021 Jul 6;23(7):e27064. doi: 10.2196/27064 (PMC8292936; doi:10.2196/27064)

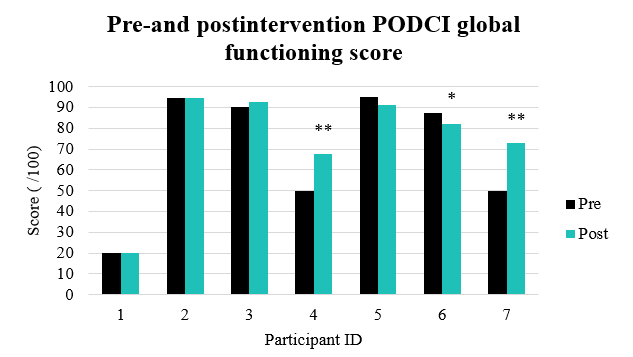

Supplement: Multimedia Appendix 3 [file jmir_v23i7e27064_app3.png]

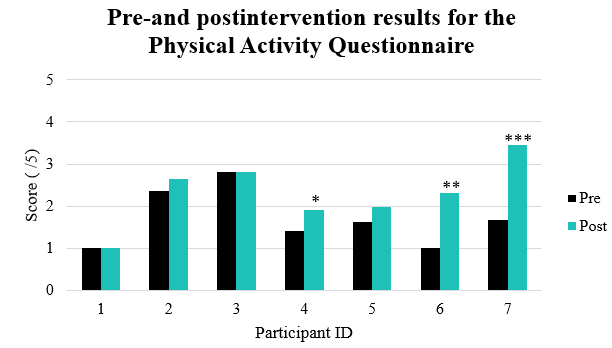

Supplement: Multimedia Appendix 4 [file jmir_v23i7e27064_app4.png]
